# How physical and sexual abuse impacts oral health: a cross-sectional study from a Norwegian Children’s House

**DOI:** 10.1186/s12903-026-09143-3

**Published:** 2026-07-02

**Authors:** Katrine Håkstad, Ewa Alicja Szyszko Hovden, Tove Irene Wigen, Liv Fegran, Unni Mette Stamnes Köpp

**Affiliations:** 1 Faculty of Health and Sport Sciences, University of Agder, Kristiansand, Norway; 2 Public dental service, The County of Agder, Kristiansand, Norway; 3 The Oral Health Centre of Expertise in East of Norway (TkØ), Oslo, Norway; 4 Department of Paediatrics, Sørlandet Hospital HF, Kristiansand, Norway; 5 Institute of Clinical Medicine, University of Oslo, Oslo, Norway; 6 Institute of Clinical Dentistry, University of Oslo, Oslo, Norway

**Keywords:** Dental caries, Child abuse, Forensic dentistry, Oral health, Oral hygiene, Toothbrushing

## Abstract

**Background:**

Although the association between neglect and an increased risk of oral disease is well established, the independent association between physical or sexual abuse and oral health has been less extensively studied.

**Aim:**

This study explored oral health condition in children suspected of being exposed to physical or sexual abuse by examining the association between abuse type and caries experience and gingival condition. It also assessed whether these associations were modified by age, sex, and ethnicity.

**Methods:**

This cross-sectional study involved 328 children, aged 1–18 years, who underwent clinical forensic dental examinations at a Norwegian Children’s House (NCH) from 2017 to 2021 (*n* = 328) due to suspected physical or sexual abuse. Data were collected from forensic dental and medical records at the NCH and included data from extra- and intra-oral examinations accompanied by conversations with the children. Negative binomial and logistic regression were performed for unadjusted, adjusted, and stratified analyses. Data are presented as ratios of means (RMs) and odds ratios (ORs), with 95% confidence intervals (CIs).

**Results:**

Sexually abused children had a higher mean number of decayed teeth (DT) (RM 1.99, 95%CI 0.99–4.02) compared with physically abused children. They also more frequently received toothbrushing assistance. When stratifying by sex and ethnicity, the results revealed that sexually abused males had a higher mean number of DT compared with physically abused males (RM 4.23, 95%CI 1.37–13.12). A similar association was observed in sexually abused Ethnic Norwegian children (RM 3.53, 95%CI 1.60–7.82). Gingival condition varied by age. Sexually abused children exhibited better gingival condition before the age of four compared with their physically abused peers, but these conditions deteriorated after the age of thirteen, where the results showed an opposite association.

**Conclusions:**

The findings indicated that oral health condition varied according to abuse type, with poorer oral health conditions among sexually abused children than those physically abused. These results, underscore the crucial role of forensic and dental professionals in identifying oral health conditions that may signal possible abuse.

**Supplementary Information:**

The online version contains supplementary material available at https://doi.org/10.1186/s12903-026-09143-3.

## Background

Child abuse refers to actions taken by a caregiver or responsible adult that directly harm or negatively affect a child’s well-being [1], even if harm is not intended [2]. Abuse is a part of the broader category of child maltreatment, which includes all forms of physical, emotional and sexual abuse, and neglect [3]. Child physical and sexual abuse represent particularly severe forms of harm involving direct bodily injury, coercion, or exploitation, often leaving severe immediate and long-term physical and psychological consequences [4]. These types of abuse are recognised as severe public health concerns [4]. The European Status Report on Preventing Child Maltreatment (2021) has estimated the prevalence of child physical abuse to be 22.9% and child sexual abuse to be 9.6% (13.4% in girls and 5.7% in boys) [5]. Children exposed to one type of abuse often experience other forms of abuse or neglect as well [6, 7]. Despite these concerning figures, many cases of child abuse may go undetected, allowing cycles of harm and trauma to persist. This can have serious implications for a child’s overall health, including oral health. These findings underscore the need to examine how child maltreatment impacts oral health, as the mouth often reveals signs of abuse.

The World Health Organization (WHO) defines oral health as “the state of the mouth, teeth and orofacial structures that enable individuals to perform essential functions, such as eating, breathing and speaking, and encompasses psychosocial dimensions, such as self-confidence and the ability to socialise and work without pain, discomfort and embarrassment” [8]. Poor oral hygiene, such as inadequate brushing and flossing routines, increases the risk of dental diseases, infections, and inflammation in the mouth, negatively affecting gingival condition, which can lead to impaired oral health [9]. Poor oral health can adversely affect children’s physical and cognitive development and consequently diminish their overall quality of life [8, 9]. To secure lifelong good oral health, initiation of toothbrushing twice daily with fluoridated toothpaste is recommended from the eruption of the first tooth [10]. Parental involvement is necessary to establish and maintain proper oral hygiene routines for young children [11]. As children transition into adolescence, the responsibility for oral hygiene gradually shifts from parents to the adolescent. In cases of child abuse, oral hygiene assistance may be compromised, leading to neglect of the child’s oral health [12, 13]. Myran et al. [14] found that exposure to traumatic experiences, including abuse, was associated with less frequent toothbrushing, which may negatively impact oral hygiene behaviours [14].

Several studies have found that children who experience abuse or neglect exhibit a higher prevalence of dental caries and an increased risk of overall poor oral health [9, 15–19]. One study has found that sexually abused children may have poorer oral health outcomes than children experiencing other abuse [20]. Other studies have reported no significant differences in caries prevalence across different types of abuse [16, 18, 19, 21]. Severe, untreated caries may indicate neglect, but the relationship between abuse type and caries remains unclear [16, 22].

The mechanisms linking child abuse to oral health are multifactorial. Physical abuse often co-occurs with neglect and dysfunctional family environments, leading to inadequate oral hygiene and limited access to dental care [11]. In contrast, sexual abuse may trigger emotional distress and stress-related behaviours that reduce motivation for self-care and impair gingival conditions [30]. Moreover, chronic stress responses and social instability can aggravate inflammation and further deteriorate oral health [9].

In Norway, efforts are made to ensure that children suspected of being exposed to physical or sexual abuse or witnesses to violence undergo facilitated interrogations at a Norwegian Children’s House (NCH) [23]. Embracing the ‘Barnahus’ model [24] as standard practice, the NCH provides a child-friendly environment where forensic interviews, medical and dental examinations, psychological assessments, and child protection interventions are conducted under one roof with regular follow-up and referral to health and welfare agencies for ongoing support in suspected cases of abuse [23]. Since 2016, trained dentists have been part of the forensic medical team at the NCH. Forensic dental examinations are conducted in accordance with a NCH protocol at the request of the prosecution in cases involving suspected orofacial signs, such as findings or injuries to the head, neck, face, ears, lips, or inside the mouth.

Toft et al. [20] analysed public dental records but did not account for key confounders [20]. In contrast, the present study utilizes forensic clinical data to provide a more detailed assessment of oral health according to abuse type. The study therefore aimed to examine the associations between abuse type and caries experience and gingival condition in children suspected of physical or sexual abuse and further assess whether these associations were modified by age, sex, and ethnicity. Based on previous research, we hypothesised that child abuse negatively affects oral health and that oral health outcomes vary according to the type of abuse experienced.

## Methods

This retrospective cross-sectional study has been prepared in accordance with the STROBE checklist [25] (Appendix B).

### Participants

**Fig. 1:**
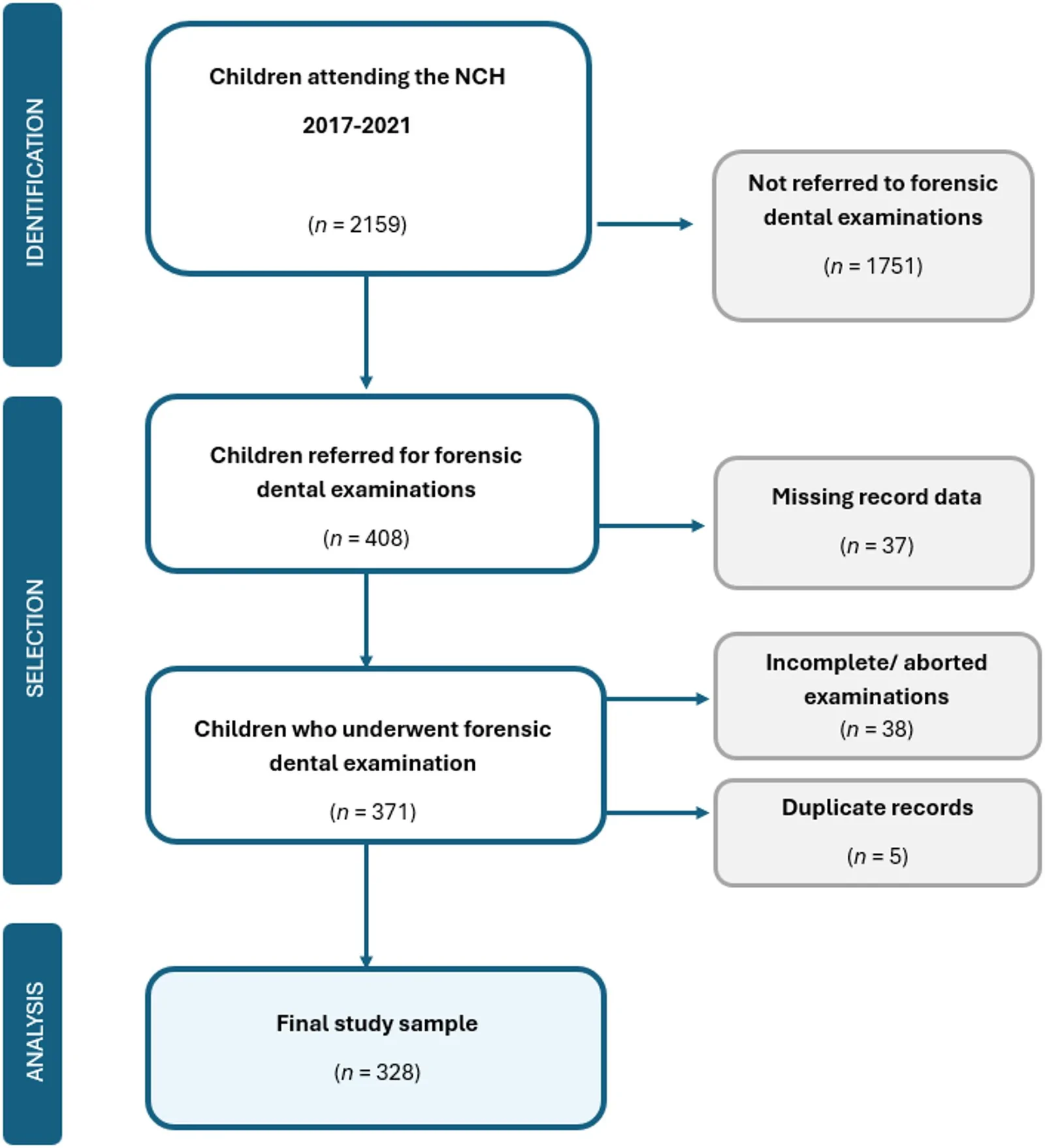
The inclusion and exclusion of participants in the final study sample (*n* = 328)

Between 2017 and 2021, 2159 children and adolescents (aged 1–18 years old) attended the Norwegian Children’s House (NCH) in Southern Norway on suspicion of physical or sexual abuse occurring within close relationships. Of these, 408 were referred to and underwent a forensic dental examination due to suspected abuse involving the orofacial region (head, face, neck, ears, lips and oral cavity Eighty cases with incomplete dental or forensic reports, missing data, or duplicate records were excluded from the analysis. The final study sample comprised 328 participants (Fig. 1).

### Data collection

Data were collected from forensic dental and medical records at the NCH. The reports included the forensic assessment protocol and the dental records (OPUS). They were based on the clinical forensic examinations as well as the forensic referral documents, which including the police report, any concern reports, and the request for forensic dental examination from the prosecution authority. Based on the dentist’s conversations with the child, self-perceived information on oral hygiene routines was retrieved from notes in the dental records.

Clinical examinations, including extra-oral and intra-oral regions, were thoroughly conducted and documented. The extra-oral examination included inspection for objective signs, findings and injuries of the head, neck, ears, face, and lips, while the intra-oral clinical dental examination was conducted using a visual-tactile approach in accordance with an NCH protocol (Appendix A). Four trained dentists performed the forensic dental examinations in a modified dental office at the NCH, which was equipped with a dental chair (HEKA Dental, Unic Line), dental mirror (CE 4 Top Dent, DAB, S-Upplands Väsby, LOT 34184), sharp probe (28775 Nordenta, Stainless CE), and a dental operating light (HEKA Dental, Unic Line) for adequate illumination. No X-rays, air-syringe or cotton rolls were available.

The dental examination collected information about the prevalence of caries, caries experience, the presence and amount of plaque accumulation, and the presence and severity of gingivitis. The visual aspect allowed the clinician to identify obvious signs of dental decay, discolouration, or structural anomalies, while the tactile component aided in detecting less visible issues such as subgingival calculus or surface irregularities on the teeth [26]. Although formal calibration procedures were not conducted, inter-examiner consistency was maintained through discussion and consensus to resolve diagnostic uncertainties.

### Variables

#### Dependent (outcome) variables

Dental caries and gingival condition were the oral health outcomes and were used as dependent variables in the analyses.

Caries was recorded in both dentitions using the tooth surfaces as unit measurements. Teeth were coded based on their condition: sound, decayed (D), missing due to caries (M), or filled (F). In the analysis, caries experience (DMFT for permanent teeth and dmft for primary teeth) and untreated caries (DT) were used as continuous (COUNT) variables. All tooth surfaces were thoroughly examined, and each tooth was classified according to its most severely affected surface using a five-graded caries scoring system, where grades 1 to 2 indicated caries confined to enamel and grades 3 to 5 indicated caries extending to dentin [27]. This five-grade visual–tactile system was summarised as DT and DMFT within the WHO framework. If visual and tactile examination revealed dentine or secondary caries, surfaces were registered as decayed.

The assessment of gingival condition included the presence of visible plaque and bleeding on probing. Plaque and gingivitis were recorded as either absent or present, and further combined into gingival condition and dichotomised into “poor” and “good”. Poor gingival condition was defined as moderate to severe amounts of visible dental plaque and/or bleeding on probing. Good gingival condition was defined as having no or little visible plaque, callus, or no bleeding on probing (gingivitis). Gingival condition was assessed using a simplified dichotomous measure.

#### Independent (exposures) variable

The type of abuse, whether suspected or confirmed, was categorised as the exposure variable, with children being classified as victims of either physical or sexual abuse the forensic reports. Sexual abuse included but was not restricted to oral sexual abuse. Information on overlapping or co-occurring abuse types was not available, as the authorities only recorded the primary abuse type that formed the basis for prosecution. Clinical forensic dental examinations were requested by the prosecutor only when the police investigation revealed suspicion of inflicted acts involving the orofacial region [27].

#### Covariates

The potential confounding factors included the demographic factors of age, sex, and parental national origin. Age was recorded as a continuous variable and divided into age groups (i.e., 1–5 years old, 6–11 years old, 12–18 years old). Sex was recorded as male or female. Parental national origin was classified as Norwegian or non-Norwegian, with non-Norwegian referring to children with one or both parents of foreign origin, regardless of their geographical background.

#### Mediators

Toothbrushing frequency, fluoride supplements and toothbrushing assistance (for children under 12 years) were self-reported oral health behaviours collected through conversations with the child during the dental examination. Toothbrushing frequency was reported as morning and evening, once a day, or seldom/never, and dichotomised as twice a day (morning and evening) and once a day or less. The use of fluoride supplements and toothbrushing assistance were reported as yes, no, or seldom/never, and dichotomised as yes and no (no and seldom/never).

#### Statistical analyses

Statistical analyses were performed using STATA version 18.0 (Stata Corp., College Station, Texas, USA) [28]. Descriptive statistics in the form of frequencies (percentages) were used to summarise categorical data, while means and standard deviations (SD) were used to summarise continuous data. Normality of continuous variables was assessed using the Shapiro-Wilk test.

The difference in mean values between children who were sexually and physically abused was assessed using independent samples t-test. Associations between categorical variables were analysed using the Chi-square test if the expected cell count was > 5. Otherwise, a Fisher’s exact test was used.

Negative binomial regression (NBR) models were chosen to investigate the association between abuse and count of caries experience and untreated caries (DT) as these variables exhibited overdispersion, making NBR more appropriate than any contrasts. Estimates of the ratio of means (RM) with 95% confidence intervals (CI) were obtained from the NBR models, showing an increase or decrease in the count of caries experience and untreated caries among the children who were sexually abused relative to those who were physically abused. Further, a binary logistic regression was used to assess the likelihood of having poor gingival condition among the children who were sexually abused compared with children who were physically abused. All statistical models were adjusted for age, sex and ethnicity, and the statistical significance level was set at a = 0.05.

Furthermore, possible interactions between abuse types and sex or ethnicity were checked. The likelihood ratio (LR) test in STATA was used to assess effect modification on models with two-way interactions to assess the associations between abuse type and caries in men separately from women and in Norwegians separately from non-Norwegians.

**Fig. 2:**
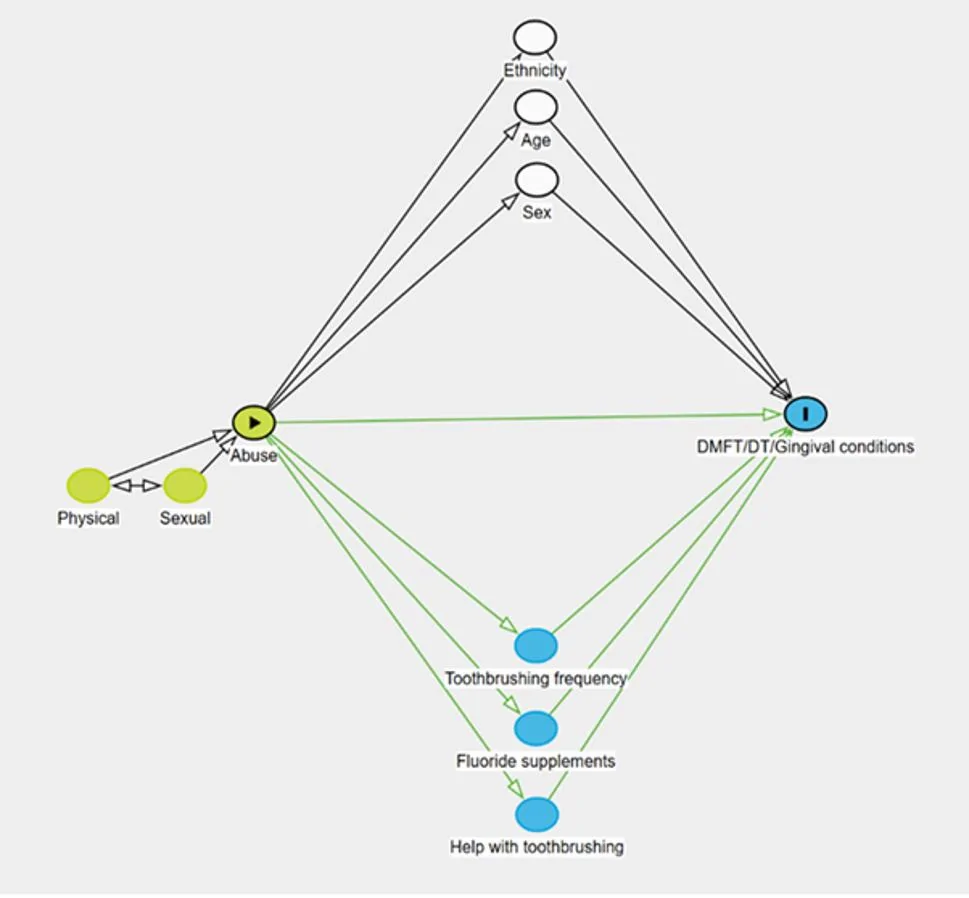
Directed acyclic graph (DAG) for hypothesised associations between exposures, outcomes, and confounders

The analytical strategy and the relationship between the variables and potential mediating pathways are described in the Directed acyclic graph (DAG; Fig. 2). In the multivariable analyses, guided by the DAG, physical and sexual abuse were considered exposure variables, while caries experience, untreated caries, and gingival condition were treated as outcome variables. Age, sex, and ethnicity were included as confounding factors. Although the DAG illustrated possible mediation pathways, we did not adjust for mediators in the models, as the goal was to assess the total effect of the relationship between the exposure and the outcomes. The DAG served as a framework to guide the data analysis strategy and to reduce the risk of bias [29].

## Results

### Characteristics of participants

Table 1 shows the characteristics of all participants according to the type of abuse. The mean age was 8.29 (SD 3.46), and more than half of the children were between 6 and 11 years of age. Sex and ethnicity were evenly distributed in both groups.

The majority of the children (86.9%) were suspected of having experienced physical abuse, while 13.1% were suspected of being exposed to sexual abuse. A higher proportion of females were suspected of experiencing sexual abuse compared with males. The occurrence of physical abuse increased with age, while sexual abuse was most common among the youngest children. Norwegian children were more often suspected of experiencing sexual abuse compared with non-Norwegian children (Table 1).

**Table 1:** Characteristics of participants according to type of abuse

	Total (*n* = 328)	Physical abuse (*n* = 285)	Sexual abuse (*n* = 43)	*p*-value
	*n* (%)	*n* (%)	*n* (%)	
Socio-demographics				
Age in years (mean ± SD)	8.29 ± 3.46	8.39 ± 3.47	7.67 ± 3.39	0.21
Age groups in years				
1–5	77 (23.5)	61 (79.2)	16 (20.8)	
6–11	187 (57.0)	165 (88.2)	22 (11.8)	0.05
12–18	64 (19.5)	59 (92.2)	5 (7.8)	
Sex				
Female	163 (49.7)	132 (81.0)	31 (19.0)	< 0.01
Male	165 (50.3)	153 (92.7)	12 (7.3)	
Ethnicity				
Norwegian	143 (43.6)	112 (78.3)	31 (21.7)	< 0.01
Non-Norwegian	185 (56.4)	173 (93.5)	12 (6.5)	

Table 2 shows oral health behaviour for all children according to type of abuse.

Approximately half of the children (52.2%) reported toothbrushing twice daily, whereas only one-third reported using fluoride supplements. Almost half of the 215 children under the age of 12 (43.3%) received parental toothbrushing assistance. A higher proportion of children who had experienced sexual abuse received toothbrushing assistance. More than one-third of the children exhibited caries experience, with no statistically significant differences observed between the different types of abuse.

**Table 2:** Oral health behaviour for children according to type of abuse

	Total (*n* = 328)	Physical abuse (*n* = 285)	Sexual abuse (*n* = 43)	*p*-value
	*n* (%)	*n* (%)	*n* (%)	
*Toothbrushing frequency				
Twice daily	145 (52.2)	127 (87.6)	18 (12.4)	0.78
Once daily or less	133 (47.8)	115 (86.5)	18 (13.5)	
*Fluoride supplements				
Yes	59 (37.1)	47 (79.7)	12 (20.3)	0.39
No	100 (28.4)	85 (62.9)	15 (15.0)	
Toothbrushing assistance (< 12 y) (*n* = 215)				
Yes	93 (43.3)	76 (81.7)	17 (18.3)	0.03
No	122 (56.7)	112 (91.8)	10 (8.2)	

*Reduced number because of internal drop-out

### Association between abuse and caries experience

Table 3 shows results from the NBR analyses exploring the association between abuse type and caries experience and untreated caries. There was no significant difference in the mean caries experience and untreated caries between children who were sexually abused and those who were physically abused. However, when adjusted for sex, age and ethnicity, the results showed that sexual abuse was associated with a higher mean number of decayed teeth (RM 1.99, 95% CI 0.99–4.02) and decayed, missed, filled teeth (RM 1.76, 95% CI 0.98–3.16) compared with those who were physically abused.

### Sex-specific associations between type of abuse and caries experience

The LR test indicated effect modification by sex. An association between caries experience and child sexual abuse compared with physical abuse was only found in males. Results showed that sexually abused males were associated with more than four times higher mean decayed teeth (RM 4.23, 95% CI 1.37–13.12) and nearly three times higher caries experience (RM 2.81, 95% CI 1.00–7.86) compared with physically abused males; however, the CIs were wide.

### Ethnicity-specific associations of sexual and physical abuse with caries experience

Results from the NBR analysis showed that when stratified by ethnicity, Norwegian children exposed to sexual abuse had three and a half times more decayed teeth (RM 3.53, 95% CI 1.60–7.82) compared with those who experienced physical abuse, and almost three times higher caries experience (RM 2.95, 95% CI 1.51–5.75; Table 3).

By contrast, non-Norwegian children who were physically abused had a significantly higher mean number of decayed, missing, and filled teeth (RM 0.27, 95% CI 0.09–0.80) compared with sexually abused non-Norwegians (Table 3).

**Table 3:** Association between type of abuse and caries (DMFT and DT) stratified by sex and ethnicity

		DMFT	Crude	Adjusted^a^	DT	Crude	Adjusted^a^
	*n* (%)	Mean and median (IQR)	RM (95% CI)	RM (95% CI)	Mean and median (IQR)	RM (95% CI)	RM (95% CI)
Total	328						
Physical abuse	285 (86.9)	1.83, 0 (0–3)	Reference	Reference	0.80, 0 (0–1)	Reference	Reference
Sexual abuse	43 (13.1)	1.93, 0 (0–4)	1.06 (0.61, 1.82)	1.76 (0.98, 3.16)	0.95, 0 (0–2)	1.19 (0.62, 2.29)	1.99 (0.99, 4.02)
Stratified by sex							
Female	163						
Physical abuse	132 (81.0)	1.90, 1 (0–3)	Reference	Reference	0.72, 0 (0–1)	Reference	Reference
Sexual abuse	31 (19.0)	1.55, 0 (0–2)	0.81 (0.43, 1.55)	1.25 (0.61, 2.55)	0.65, 0 (0–1)	0.90 (0.39, 2.08)	1.19 (0.47, 2.98)
Male	165						
Physical abuse	153 (92.7)	1.76, 0 (0–2)	Reference	Reference	0.87, 0 (0–1)	Reference	Reference
Sexual abuse	12 (7.3)	2.91, 0.5 (0–5.5)	1.65 (0.60, 4.55)	2.81 (1.00, 7.86)	1.75, 1 (0–2.5)	2.01 (0.67, 6.07)	4.23 (1.37, 13.12)
Stratified by ethnicity							
Norwegian	143						
Physical abuse	112 (78.3)	0.93, 0 (0–1)	Reference	Reference	0.38, 0, (0–0)	Reference	Reference
Sexual abuse	31 (21.7)	2.42, 1 (0–5)	2.61 (1.32, 5.16)	2.95 (1.51, 5.75)	1.19, 0 (0–2)	3.18 (1.44, 7.01)	3.53 (1.60, 7.82)
Non-Norwegian	185						
Physical abuse	173 (93.5)	2.41, 1 (0–3)	Reference	Reference	1.08, 0, (0–1)	Reference	Reference
Sexual abuse	12 (6.5)	0.67, 0 (0–1)	0.28 (0.10, 0.80)	0.27 (0.09, 0.80)	0.33, 0, (0–0)	0.31 (0.08, 1.21)	0.36 (0.09, 1.40)

*IQR,* interquartile range, *RM,* ratio of means, *CI,* confidence interval, *DMFT,* decayed, missed, filled, teeth, *DT,* decayed teeth. ^a^ adjusted for age (continuous), sex (male/female) and ethnicity (Norwegian/Non-Norwegian)

**Fig. 3:**
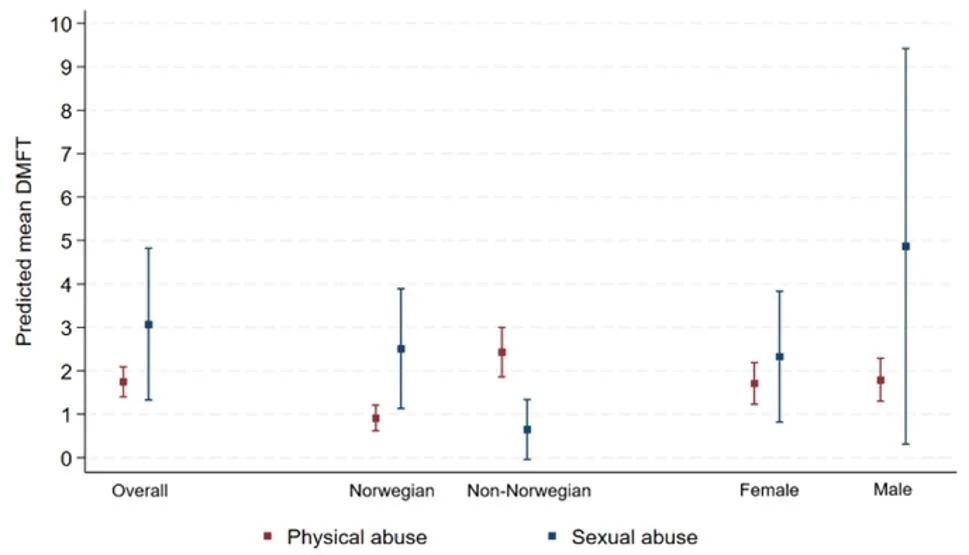
Predicted mean DMFT scores among physically and sexually abused children by ethnicity and sex. Non-Norwegian children who experienced physical abuse had significantly higher mean DMFT scores than Norwegian children, as indicated by non-overlapping error bars

**Fig. 4:**
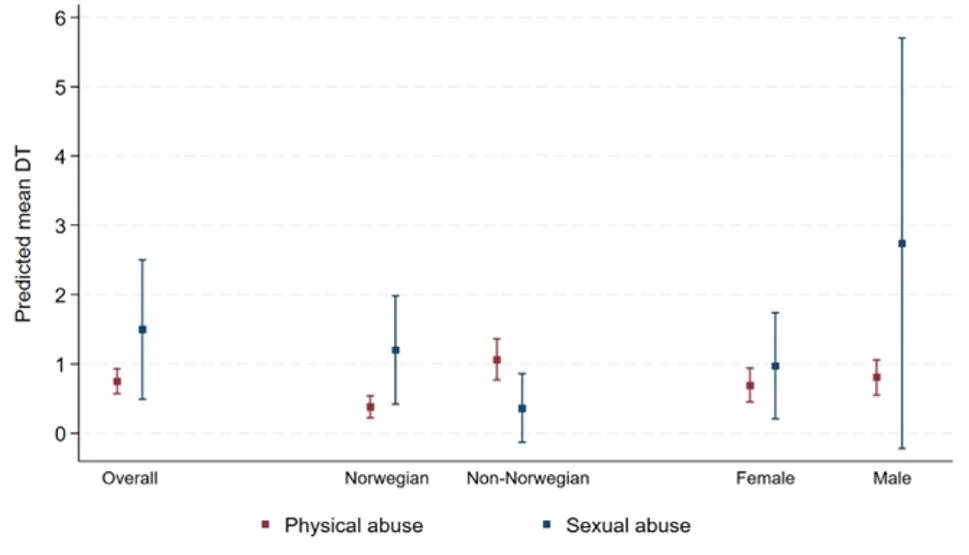
Predicted mean DT scores among physically and sexually abused children by ethnicity and sex. Non-Norwegian children who experienced physical abuse had significantly higher mean DT scores than Norwegian children, as indicated by non-overlapping error bars

Figure 3 and 4 shows that the predicted mean number of decayed teeth and caries experience in children who were physically abused was higher among non-Norwegian children compared with Norwegian children. Results of the regression analyses illustrated how sexual abuse correlated with caries across stratified groups, sex and ethnicity compared with physical abuse.

### Association between abuse type and gingival condition

**Fig. 5:**
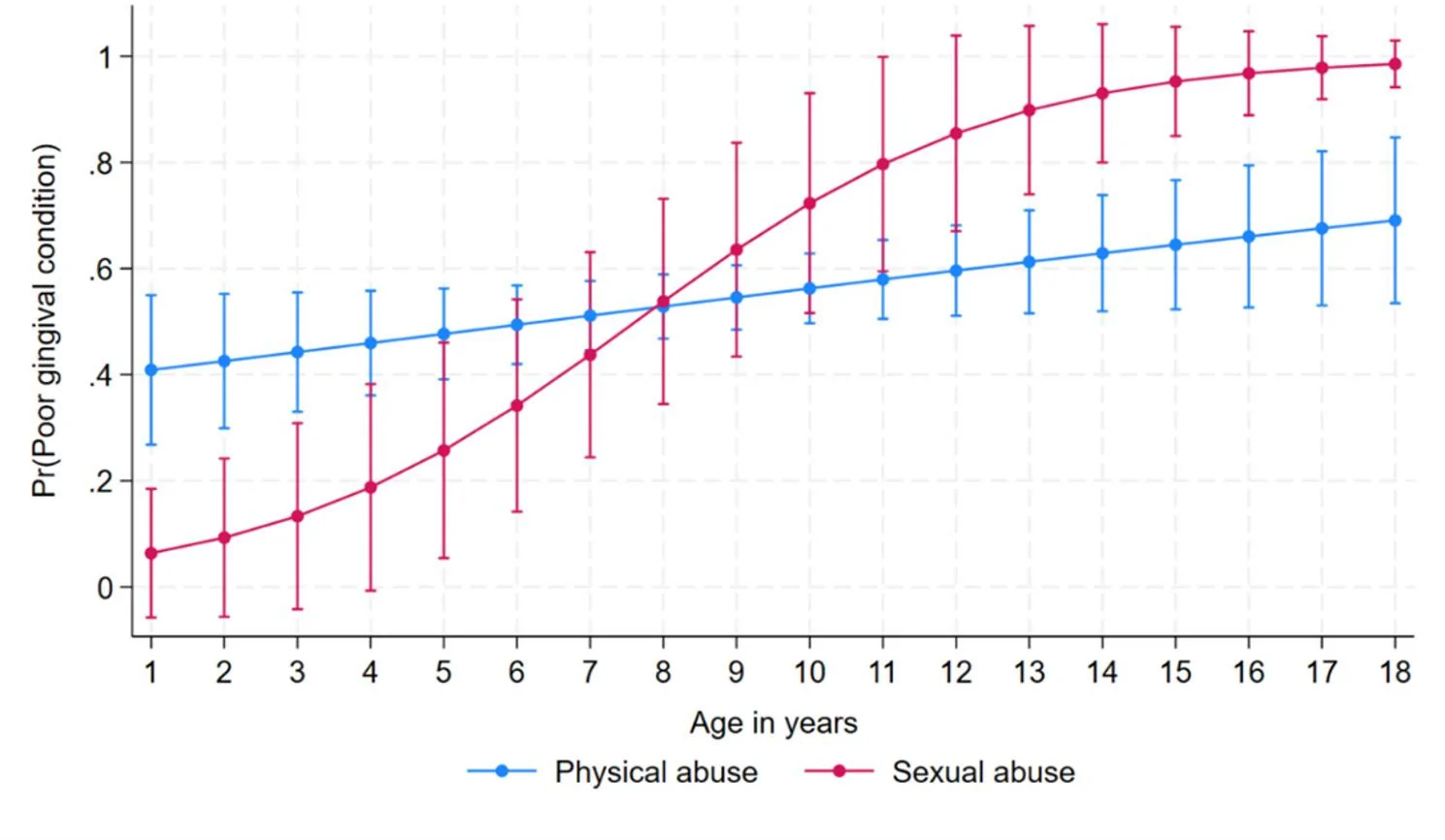
Differences in gingival conditions from childhood to adulthood and the predicted probability of poor gingival conditions across the age span of 1–18 years in children who experienced physical and sexual abuse

Table 4 shows the relationship between the different types of abuse and gingival condition in the study sample, categorised by sex and ethnicity. Overall, the odds of having poor gingival condition did not differ between children who experienced sexual abuse and those who experienced physical abuse (Table 4). However, children under the age of four years who were sexually abused had significantly better gingival condition compared with physically abused children, a trend that reversed by the age of eight. From the age of 13 years, the likelihood of having poor gingival condition was higher among the children who were sexually abused than those who were physically abused (Fig. 5). Figure 5 illustrates the likelihood of having poor gingival condition across ages when exposed to child physical or sexual abuse.

**Table 4:** Association between type of abuse and gingival condition stratified by sex and ethnicity

	Total (*n* = 305)	Gingival cond. Prevalence poor	Crude	Adjusted^a^
	*n* (%)	*n* (%)	OR (95% CI)	OR (95% CI)
Physical abuse	267(87.5)	145 (54.3)	Reference	Reference
Sexual abuse	38 (12.5)	17 (44.7)	0.68 (0.34, 1.35)	0.95 (0.46, 1.98)
Stratified by sex				
Female	150			
Physical abuse	121 (80.7)	59 (48.8)	Reference	Reference
Sexual abuse	29 (19.3)	14 (48.3)	0.98 (0.44, 2.20)	1.17 (0.51, 2.74)
Male	155			
Physical abuse	146 (942)	86 (58.9)	Reference	Reference
Sexual abuse	9 (5.8)	3 (33.3)	0.35 (0.08, 1.45)	0.57 (0.13, 2.58)
Stratified by ethnicity				
Norwegian	132			
Physical abuse	104 (78.8)	52 (50.0)	Reference	Reference
Sexual abuse	28 (21.2)	10 (35.7)	0.56 (0.23, 1.32)	0.75 (0.30, 7.88)
Non-Norwegian	173			
Physical abuse	163 (94.2)	93 (57.1)	Reference	Reference
Sexual abuse	10 (5.8)	7 (70.0)	1.76 (0.44, 7.03)	2.04 (0.49, 8.44)

*OR*: odds ratio, *CI*: confidence interval

^a^Adjusted for age (continuous), sex (male/female) and ethnicity (Norwegian/Non-Norwegian)

Gingival condition (*n* = 305) categorised as healthy or poor (plaque/callus/gingivitis), 23 children with missing data/answers were excluded

## Discussion

This study examined the impact of physical and sexual abuse on oral health in children at a Norwegian Children’s House. The findings support the hypothesis that child abuse negatively affects oral health with outcomes varying by abuse type.

The results showed that children who had a history of sexual abuse demonstrated higher mean DT and DMFT scores and more often received toothbrushing assistance compared with children who had experienced physical abuse. Differences in caries prevalence between children suspected of sexual versus physical abuse may stem from variations in caregiving environments and stress-related behaviours [9, 30]. The abuse in this cohort occurred within close relationships, and in most cases the suspected perpetrator was the primary caregiver or a person close to the family. This relational proximity may contribute to both emotional distress and reduced attention to daily oral hygiene routines. Several studies confirmed an association between poor oral health, particularly caries prevalence [14, 16, 18], and child maltreatment [9, 15, 16, 18, 21] or neglect [17, 31]. Nakamura et al. [16] described a difference in caries prevalence among abused children, with an odds ratio of 4.1, but only an association with neglect was specified and not the specific abuse types [16].

To our knowledge, only one study has investigated the association between oral health in sexually abused children versus physically abused children using data from dental records of children referred to an NCH [20]. Our findings align with results from that study, which reported four times higher caries prevalence among children suspected of experiencing sexual abuse compared with those suspected of experiencing physical abuse [20]. However, unlike that study, the present study provided a more accurate assessment of the relationship between abuse type and oral health by controlling for key confounders, such as age, sex, and ethnicity. This finding suggests that the present study may shed light on a less-examined aspect of the relationship between abuse and oral health, particularly the higher mean number of decayed teeth among males, warranting further investigation. Moreover, our results on the specific association between oral health outcomes and cases of physical abuse versus sexual abuse remain relatively underexplored, with limited research on this comparison.

An unexpected finding was the higher levels of decayed teeth in sexually abused Norwegian children compared with their physically abused counterparts, alongside a greater caries prevalence in physically abused non-Norwegian children compared with physically abused Norwegian children. The increased caries prevalence in children with parents of non-Norwegian origin aligns with the results of a scoping review by Folayan et al. [31] that examined childhood caries in individuals with a migrant background [31]. However, they did not differ between abuse types. Our findings may indicate that ethnicity plays an important moderating role in the relationship between the type of abuse and oral health outcomes. This observation may be further explained by cultural barriers to healthcare, stigma surrounding sexual abuse, or health-related factors unique to these populations [19, 31]. These patterns may indicate complex interactions between ethnicity, type of abuse, and oral health, meriting further investigation and potentially providing valuable insights into the relationship between child abuse and health disparities.

This study revealed age-specific variations in gingival condition. Sexually abused children exhibited better gingival condition before the age of four, followed by a decline after the age of eight, with gingival health worsened by adolescence in sexually abused children compared with their physically abused counterparts. To our knowledge, these associations have not been previously documented. The age-related decline in gingival health may partly reflect the transition from caregiver-assisted toothbrushing in early childhood to increasing self-responsibility in adolescence, when motivation and oral hygiene routines often weaken [11, 12]. The observed association between abuse type and gingival condition may arise through several mechanisms. Physical abuse could directly affect orofacial regions or indirectly lead to poor oral hygiene through dental neglect [7], while sexual abuse might influence gingival conditions via stress-mediated pathways or reduced motivation for self-care [17]. Moreover, a proportion of the included children had parents originating from countries where corporal punishment is not legally prohibited, and such cultural differences in caregiving norms and health behaviours may have influenced both oral hygiene practices and stress responses [31, 32]. Nevertheless, the cross-sectional design and relatively small subgroups limit inference, and the observed differences may partly be due to random variation. Further research is needed to confirm these associations.

Parental involvement is essential for young children’s oral health outcomes [10–13]. Compromised caregiving can lead to insufficient supervision and reduced toothbrushing frequency among abused children, thereby increasing the risk of adverse oral health outcomes [10]. Within the NCH cohort, among children under twelve years who received toothbrushing assistance, those who had experienced sexual abuse received assistance more frequently than those who had experienced physical abuse—a somewhat surprising finding [32]. The distinction between oral and non-oral sexual abuse may be relevant in this context, as oral abuse could have a more direct impact on oral health behaviours. This possible association contrasts with previous research in children aged 2–18 years old, which has generally shown that abused children typically receive less preventive dental care [16]. Additionally, Matsuyama et al. [11] demonstrated that insufficient parental involvement and lack of supervision were associated with a higher prevalence of caries and unhealthy oral health behaviours [11]. Beyond caregiving factors, psychosocial mechanisms such as fear, avoidance, and disrupted daily routines may also contribute to poorer oral hygiene among abused children by reducing motivation for self-care and regular dental attendance [14, 30]. These findings contribute to understanding the complex relationship between oral hygiene, toothbrushing behaviour, and parental involvement.

Childhood sexual abuse has been linked to psychosocial and behavioural problems in adulthood, resulting in increased emotional distress and challenges in maintaining self-care routines, including oral hygiene [14, 30]. In contrast, although childhood sexual abuse is often linked to re-traumatisation and dental anxiety in adulthood, such responses are less commonly observed in children [30]. This discrepancy may result from children’s ability to conceal psychological distress and stigma, making abuse more challenging to identify through behaviour or verbal communication alone [30]. Their ability to mask symptoms emphasises the need for comprehensive health assessments, including dental examinations, to identify potential abuse [30].

All studies have some strengths and limitations. A major strength of this study is the use of data from clinical examinations of children with police-reported abuse. Unlike most existing studies comparing abused with non-abused children, this research provides valuable insights into comparisons between abuse types. Moreover, the study uniquely controlled for confounding factors like age, sex, and ethnicity in this demographic. However, the study’s findings should be interpreted considering some limitations. Abuse types were categorised based on the primary reason for the criminal investigation as either physical or sexual abuse. However, the data did not include information on whether oral sexual abuse had occurred. Furthermore, different types of abuse often overlap and should be considered in cases where multiple types coexist. Ethnicity was categorised differently in this study than in others, which may have influenced the observed variations related to ethnic differences. Data were collected from a single Children’s House in one region of Norway, and information on parental education and socioeconomic status was not available. The absence of these covariates may contribute to residual confounding and limits the generalisability of the findings. Dentists had access to the forensic referral, including the police report, concern reports, and requests for forensic dental examination from the prosecution authority, but external dental records were not available; therefore, comparison with previous or external dental treatment data was not possible.

Although orofacial injuries are frequently observed in abused children and were assessed within this research project, these findings were not included in the present analysis, as they are being examined in detail in a separate study based on the same forensic dataset.

The protocol was based on national guidance and WHO/DMFT standards and was internally piloted but not formally validated [27]. Furthermore, using visual-tactile dental examinations without X-rays restrict the ability to detect interproximal, subgingival, or early caries and periapical conditions, increasing the risk of underdiagnosing oral health disease. This study evaluated DMFT for both primary and permanent dentitions as a measure of total caries experience and does not account for differences in dentitions type or stages of dental development. Lastly while dental caries and gingival condition were assessed, intra-oral soft tissue signs of abuse were not evaluated in this study. Furthermore, gingival condition was assessed using a simplified dichotomous measure based on visible plaque and bleeding on probing rather than a standardized clinical index, which may limit measurement validity and comparability with other studies.

## Conclusion

This study demonstrated distinct oral health disparities in sexually abused children compared with those physically abused, emphasising the importance of incorporating oral health assessments into forensic evaluations of suspected abuse. The results reinforce the important role of forensic and dental professionals in recognising oral health conditions that may signal child abuse, including, but not limited to, neglect. Future research should further explore the differential impact of different types of abuse on oral health. Moreover, enhancing awareness and knowledge among dental professionals and healthcare personnel through strengthened protocols for detection, documentation, and follow-up is important to improve the identification, appropriate referral and management of child abuse.

## Supplementary Information

Supplementary Material 1.

Supplementary Material 2.

Supplementary Material 3.

## Data Availability

The datasets generated and analysed during the current study are not publicly available. Access to these data is restricted due to privacy concerns and ethical considerations, especially given the sensitive forensic and legal context of the study. Therefore, the data cannot be shared publicly and are available only upon reasonable request, subject to appropriate legal and ethical approvals.

## Abbreviations

NCH: Norwegian Children’s House
DAG: directed acyclic graph
NBR: negative binomial regression
SD: standard deviation
DMFT: decayed, missed, filled teeth
DT: decayed teeth/untreated caries
RMs: ratios of means
Cis: 95% confidence intervals
ORs: odds ratios

## References

[1] World Health Organization. Child maltreatment. World Health Organization. 2024. http://www.who.int/news-room/fact-sheets/detail/child-maltreatment. Accessed 25 Nov 2024.

[2] Leeb RT. Child maltreatment surveillance: uniform definitions for public health and recommended data elements. Centers for Disease Control and Prevention, National Center for Injury Prevention and Control. 2008.

[3] Gonzalez D, Bethencourt Mirabal A, McCall JD. Child Abuse and Neglect. Treasure Island (FL): StatPearls Publishing; 2025 Jan-. Available from: https://www.ncbi.nlm.nih.gov/books/NBK459146/. 29083602

[4] Pekarsky AR. Overview of Child Maltreatment (child abuse; child neglect). MSD MANUAL, Professional Version. 2022. Available from: https://www.msdmanuals.com/professional/pediatrics/child-maltreatment/overview-of-child-maltreatment. Acccessed 20 May 2024.

[5] Sethi DBM, BM, Hughes K, Gilbert R, Mitis F, Galea G. European report on preventing child maltreatment. Copenhagen: WHO Regional Office for Europe; 2013. https://mzd.gov.cz/wp-content/uploads/wepub/8350/18822/ERPCM_finalreport_web.pdf. Accessed 12 Apr 2024.

[6] Hafstad GS, Augusti E-M. Ungdoms erfaringer med vold og overgrep i oppveksten [Adolescents’ experiences with violence and abuse during upbringing]. Oslo: Nasjonalt kunnskapssenter om vold og traumatisk stress; 2019. https://www.nkvts.no/content/uploads/2019/10/Rapport_4_19_UEVO.pdf. Accessed 10 Mar 2024.

[7] Håkstad K, Fegran L, Hovden E, Köpp UMS. Orofacial signs of child or adolescent maltreatment identified by dentists and dental hygienists: A scoping review. Int J Paediatr Dent. 2024;34(3):285–301. 10.1111/ipd.13139 38050876

[8] World Health Organization.Global oral health status report: towards universal health coverage for oral health by 2030. Geneva: World Health Organization. 2022. Available from: https://www.who.int/publications/i/item/9789240061484. Accessed 18 Feb 2024.

[9] Peres MA, Macpherson LMD, Weyant RJ, Daly B, Venturelli R, Mathur MR, Listl S, Celeste RK, Guarnizo-Herreño CC, Kearns C, et al. Oral diseases: a global public health challenge. Lancet. 2019;394(10194):249–60. 10.1016/S0140-6736(19)31146-8 31327369

[10] Kumar S, Tadakamadla J, Johnson NW. Effect of Toothbrushing Frequency on Incidence and Increment of Dental Caries: A Systematic Review and Meta-Analysis. J Dent Res. 2016;95(11):1230–6. 10.1177/0022034516655315 27334438

[11] Matsuyama Y, Isumi A, Doi S, Fujiwara T. Poor parenting behaviours and dental caries experience in 6- To 7‐year‐old children. Community Dent Oral Epidemiol. 2020;48(6):493–500. 10.1111/cdoe.12561 PMC7689935 32750206

[12] Zaborskis A, Kavaliauskienė A, Levi S, Tesler R, Dimitrova E. Adolescent Toothbrushing and Its Association with Sociodemographic Factors—Time Trends from 1994 to 2018 in Twenty Countries. Healthcare. 2023;11(24):3148. 10.3390/healthcare11243148 PMC10742657 38132038

[13] Yıldız G, Ceyhan D. Knowledge, awareness, attitudes and experiences of parents of dental patients regarding child abuse and neglect. BMC Oral Health. 2025;25(1):305. 10.1186/s12903-025-05647-6 PMC11863918 40011915

[14] Myran L, Sen A, Willumsen T, Havnen A, Kvist T, Rønneberg A, Dahllöf G, Høvik H. Associations of adverse childhood experiences with caries and toothbrushing in adolescents. The Young-HUNT4 Survey. BMC Oral Health. 2023;23(1):760. 10.1186/s12903-023-03492-z PMC10576322 37838651

[15] Duda JG, Biss SP, Bertoli FM, Bruzamolin CD, Pizzatto E, Souza JF, Losso EM. Oral health status in victims of child abuse: a case-control study. Int J Paediatr Dent. 2017;27(3):210–6. 10.1111/ipd.12254 27434332

[16] Nakamura Y, Nogami Y, Iwase Y, Hozawa M, Sotome T, Saitoh I, Ohuchi A, Hayasaki H. Dental caries prevalence in children during temporary protective care according to type of abuse. BMC Public Health. 2024;24(1):1345. 10.1186/s12889-024-18833-y PMC11102624 38762490

[17] Kvist T, Annerbäck EM, Dahllöf G. Oral health in children investigated by Social services on suspicion of child abuse and neglect. Child Abuse Negl. 2018;76:515–23. 10.1016/j.chiabu.2017.11.017 29294446

[18] Nogami Y, Iwase Y, Kagoshima A, Saitoh I, Nakajima T, Takahashi H, Nakagawa K, Yoshihara A, Ohuchi A, Asahito T, et al. Dental caries prevalence and treatment level of neglected children at two child guidance centers. Pediatr Dent J. 2017;27(3):137–41.

[19] Bradbury-Jones C, Isham L, Morris AJ, Taylor J. The Neglected Relationship Between Child Maltreatment and Oral Health? An International Scoping Review of Research. Trauma Violence Abuse. 2021;22(2):265–76. 10.1177/1524838019841598 31043121

[20] Toft J, Myhre AK, Sun YQ, Willumsen T, Ronneberg A. Oral health history in children referred to a child advocacy center in Norway. Child Abuse Negl. 2022;132:105789. 10.1016/j.chiabu.2022.105789 35849872

[21] Valencia-Rojas N, Lawrence HP, Goodman D. Prevalence of early childhood caries in a population of children with history of maltreatment. J Public Health Dent. 2008;68(2):94–101. 10.1111/j.1752-7325.2007.00077.x 18248339

[22] World Health Organization. Global oral health strategy. Geneva: World Health Organization. 2022. Available from: https://www.who.int/team/noncommunicable-diseases/global-status-report-on-oral-health-2022. Accessed 18 Feb 2024.

[23] Rønneberg A, Hovden ES, Bie TMG, Willumsen T, Köpp UMS. Klinisk rettsodontologiske undersøkelser ved Statens Barnehus - veier til tverrprofesjonell samhandling til barnets beste [Clinical forensic oral examination at «Statens Barnahus» - ways of cross-professional interaction in the best interest of the child]. Nor Tannlegeforen Tid. 2019;129:884–92.

[24] PROMISE Barnahus Network. Retrieved December 28. About barnahus. 2021. Available from: https://www.barnahus.eu/en/about-barnahus/Accessed.

[25] STROBE Statement. Strengthening the Reporting of Observational Studies in Epidemiology (STROBE) guidelines. 2023;20. Available from: https://www.strobe-statement.org/Accessed.

[26] Gomez J. Detection and diagnosis of the early caries lesion. BMC Oral Health. 2015;15(1):S3. 10.1186/1472-6831-15-S1-S3 PMC4580848 26392124

[27] Amarante E, Raadal M, Espelid I. Impact of diagnostic criteria on the prevalence of dental caries in Norwegian children aged 5, 12 and 18 years. Community Dent Oral Epidemiol. 1998;26(2):87–94. 10.1111/j.1600-0528.1998.tb01933.x 9645401

[28] StataCorp. Stata Statistical Software: Release 18. College Station. TX: StataCorp LLC.; 2023.

[29] Akinkugbe AA, Sharma S, Ohrbach R, Slade GD, Poole C. Directed Acyclic Graphs for Oral Disease Research. J Dent Res. 2016;95(8):853–9. 10.1177/0022034516639920 PMC4935832 27000052

[30] Søftestad S, Kranstad V, Fredriksen TV, Willumsen T. Invading Deeply into Self and Everyday Life: How Oral Health-Related Problems Affect the Lives of Child Sexual Abuse Survivors. J Child Sex Abus. 2020;29(1):62–78. 10.1080/10538712.2019.1682096 31692414

[31] Folayan MO, Schroth RJ, Ayouni I, Nguweneza A, Arheiam A, Al-Batayneh OB, Virtanen JI, Gaffar B, Duangthip D, Sun IG, et al. A scoping review linking early childhood caries to violence, neglect, internally displaced, migrant and refugee status. BMC Oral Health. 2023;23(1):747. 10.1186/s12903-023-03459-0 PMC10568772 37821894

[32] Kloppen K, Haugland S, Svedin CG, Mæhle M, Breivik K. Prevalence of Child Sexual Abuse in the Nordic Countries: A Literature Review. J Child Sex Abus. 2016;25(1):37–55. 10.1080/10538712.2015.1108944 26809050

