# Supplementary material for: How physical and sexual abuse impacts oral health: a cross-sectional study from a Norwegian Children’s House
Source: BMC Oral Health. 2026 Jul 2;26:1841. doi: 10.1186/s12903-026-09143-3 (PMC13617796; doi:10.1186/s12903-026-09143-3)
Supplement: Supplementary file 1 [file 12903_2026_9143_MOESM1_ESM.pdf]

Dato og klokkeslett for tannlegeundersøkelsen
Dato Klokkeslett

Klinisk rettsodontologisk tannlegeerklæring

Navnet på barnet/ungdommen: *Skriv tekst her*
Født: *Skriv tekst her*
Adresse: *Skriv tekst her*

Journal skrevet på anmodning fra politiet skal formuleres slik at den kan leses uten kjennskap til medisinske faguttrykk.

- Les bakgrunnsopplysningene.
- Vurder barnets alder og start med å forklare barnet hva som skal skje (hele forløpet). Vis hvilke instrumenter som skal benyttes.
- Dersom mistenkte følger barnet bør man tilstrebe og foreta undersøkelsen med annet personale tilstede.

A. Generell helsestatus

Generell informasjon er gitt av:

☐ Foreldre ☐ Mor ☐ Far ☐ Beredskapshjem/fosterhjem ☐ Barnet/ungdom

Daglige rutiner:

Er det tilkommet en endring av daglige rutiner? (spisemønster/vansker, tannpuss etc.)

☐ ua ☐ Ja, spesifiser

Rutiner på tannpuss

☐ Morgen ☐ Kveld ☐ Morgen og kveld ☐ Sjelden ☐ Aldri

Hjelp til tannpuss

☐ Nei ☐ Ja, *Spesifiser*

Problemer med tannpuss

☐ Nei ☐ Ja, *Spesifiser*

Fluorbruk

☐ Nei ☐ Ja ☐ Vet ikke

© Statens Barnehus Kristiansand

Nåværende sykdommer (astma, diabetes.....) ☐ ua ☐ Ja, *Spesifiser*

Kosthold (spesielt kosthold/diett, annet...) ☐ ua ☐ Ja, *Spesifiser*

Spiseforstyrrelse ☐ ua ☐ Ja, *Spesifiser*

Psykiske lidelse ☐ ua ☐ ADHD ☐ annet *Spesifiser*

☐ Røyk ☐ Snus ☐ Rusmidler *Spesifiser*

Tidligere sykdommer/symptomer

Astma, annet..	☐ua ☐ Ja, *Spesifiser*
Allergier	☐ua ☐ Ja, *Spesifiser*
Blødninger i tannkjøttet	☐ua ☐ Ja, *Spesifiser*
Ofte sår i munnen	☐ua ☐ Ja, *Spesifiser*
Dårlig ånde	☐ua ☐ Ja, *Spesifiser*
Ømme tyggemuskler	☐ua ☐ Ja, *Spesifiser*
Tanngnissing	☐ua ☐ Ja, *Spesifiser*
Fingersuger	☐ua ☐ Ja, *Spesifiser*
Tannbehandlingsangst/vegring	☐ua ☐ Ja, *Spesifiser*
Medikamentbruk	☐ua ☐ Ja, *Spesifiser*
Annet	☐Ja, *Spesifiser*

Komplikasjoner ved tidligere tannbehandling

☐ ua ☐ Ja, *Spesifiser*

Tannlegens vurdering av helsetilstand (ASA: American Society of Anesthestiology)

☐ ASA1 (Frisk)

☐ ASA2 (Mild system sykdom)

☐ ASA3 (Alvorlig system sykdom)

© Statens Barnehus Kristiansand

☐ ASA4 (Alvorlig systemsykdom som er konstant livstruende for liv og helse)

Dersom du har tilgang til elektronisk dokumentasjon i Nextsys eller Opus kan dette benyttes. Hvis ikke benytt punktene A og B. Generell helsestatus gir veiledende informasjon.

Klinsk rettsodontologisk tannlegeundersøkelse (ekstra og intraoral)

Hode, hals området og hele munnhulen (inspeksjon med to speil) undersøkes i godt lys med henblikk på:

- Tidligere eller aktuell sykdom som kan ha påvirkning forhold i hode, hals området eller tannstatus
- Funn som avviker fra forventet normal anatomi

Hår avrivning	☐ua	☐ Ja, *Spesifiser*
Ører og området bak ørene	☐ua	☐ Ja, *Spesifiser*
Hud (misfarging/sår..)	☐ua	☐ Ja, *Spesifiser*
Kjeve/gapefunksjon	☐ua	☐ Ja, *Spesifiser*
Lepper	☐ua	☐ Ja, *Spesifiser*
Tunge, frenulum	☐ua	☐ Ja, *Spesifiser*
Slimhinneforandringer	☐ua	☐ Ja, *Spesifiser*
Bittforhold	☐ua	☐ Ja, *Spesifiser*
Tenner med patologisk mobilitet	☐ ua	☐ Ja, *Spesifiser*
Tannfraktur (aktuelle/eldre)	☐ ua	☐ Ja, *Spesifiser*
Tidlig tannfelling	☐ ua	☐ Ja, *Spesifiser*
Gingivale forhold (plaque, gingivitt)	☐ ua	☐ Ja, *Spesifiser*
Tannslitasje/syreskader	☐ ua	☐ Ja, *Spesifiser*
Karies	☐ Ingen klinisk synlig	
	☐ Ja ☐ Moderat ☐ Grav	

- Tannlegens vurdering av munnhygiene

☐ God ☐ Middels ☐ Dårlig

Statens Barnehus Kristiansand
Besøksadresse: Vestre Strandgate 26, 4611 Kristiansand
Postadresse: Postboks 135, 4662 Kristiansand
Tlf.: +47 99 25 71 61
Faks: +47 38 61 19 00
E-post: kristiansand@barnehuset.com
Foretaksnr.: NO 983 998 275
www.statensbarnehus.no

© Statens Barnehus Kristiansand

B. Sporsikring

- I de akutte og alvorligste sakene skal sporsikring foretas i samarbeid med politiets kriminalteknikere.
- Bruk godkjent utstyr for undersøkelsen og sporsikringen (sporsikringspakke).
- Ved undersøkelsen bør man se etter: Biologisk materiale, bakterie-/virus-infeksjoner, andre kriminaltekniske spor og situasjonsspor fra handlingen.
- Hver type observasjon/funn, fremmed materiale (flekker, smuss, løse hår eller fibre)/skader/arr/sykdomstegn/merker etter behandling/prøvetaking beskrives (*ikke tolking*) hver for seg i rapporten.
- Merk fortløpende funn av med nummer på skissen.
- Beskrift poser med sportype/lokalisasjon (for eksempel munn), hva som sikres (mulig sæd, spytt etter kyssing etc.), metode for sikring (f.eks. 2 pinner gnis på inn og utside nedre tannrekke), metode for pakking (pinner fra samme lokalisasjon i merket pose).
- Prøvetaking og håndtering av prøvemateriale skjer i samråd med politiet.

C. Dokumentasjon

- Tegning av skisser nummereres fortløpende fra 1 og utover – se vedlegg
- Røngten funn
- Foto/videodokumentasjon (foretatt av hvem?)
- Eventuelt vedlegg fra elektonisk pasient journal (EPJ)

D. Kopi av journal fra tidligere legebesøk/behandling

- Ved utskrift fra av annen/tidligere journal bør faguttrykk forklares

E. Tannlegeerklæring basert på undersøkelse i henhold til punkt A + B eller D + evt B hvis sporsikring er utført.

Statens Barnehus Kristiansand
Besøksadresse: Vestre Strandgate 26, 4611 Kristiansand
Postadresse: Postboks 135, 4662 Kristiansand

© Statens Barnehus Kristiansand

Tlf.: +47 99 25 71 61
Faks: +47 38 61 19 00
E-post: kristiansand@barnehuset.com
Foretaksnr.: NO 983 998 275
www.statensbarnehus.no

- Vurder å starte med en kort oppsummering av kjente/mistenkte forhold i saken (finnes det flere versjoner bør alle nevnes)
- Beskriv barnet generelt og i undersøkelsessituasjonen (allmenntilstand, vegring, usedvanlig brekningsrefleks, underkastelse)
- Beskrivelse av funn under punkt A
- Kort sammenfatning av relevante undersøkelsesfunn under punkt B (tolking, ikke ny beskrivelse)
    - Ferske skader
    - Eldre skader
    - Sykelige forandringer etc.
    - Tegn på beruselse/påvirkning
    - Evt. prøveresultat/analyseresultat
    - Evt. annet av relevans
- Hvordan har skaden oppstått (type av vold)
- Hvor gamle er skadene (timer/dager/uker/måneder)
- Drøft
    - Kan skadene ha oppstått i det oppgitte tidsrom som angitt i anmeldelsen?
    - Stemmer skadebildet med det hendelsesforløpet og den tid som er angitt i bakgrunnsinformasjonen? (finnes det flere versjoner bør alle kommenteres)
    - Stemmer det ikke – tyder skadebildet på annen skademekanisme/annet tidspunkt?
    - Eventuelt tyder skadebildet på at skadene er påført av den skadede selv eller annen person i forståelse med den skadede?
    - Er skadene av livstruende alvorlighetsgrad?
    - Medfører skadene risiko for mén?

Statens Barnehus Kristiansand
Besøksadresse: Vestre Strandgate 26, 4611 Kristiansand
Postadresse: Postboks 135, 4662 Kristiansand

Tlf.: +47 99 25 71 61
Faks: +47 38 61 19 00
E-post: kristiansand@barnehuset.com
Foretaksnr.: NO 983 998 275
www.statensbarnehus.no

© Statens Barnehus Kristiansand

STATENS BARNEHUS
Et tverrfaglig kompetansehus
Kristiansand

Dokumentasjon/vedlegg til hovedrapporten

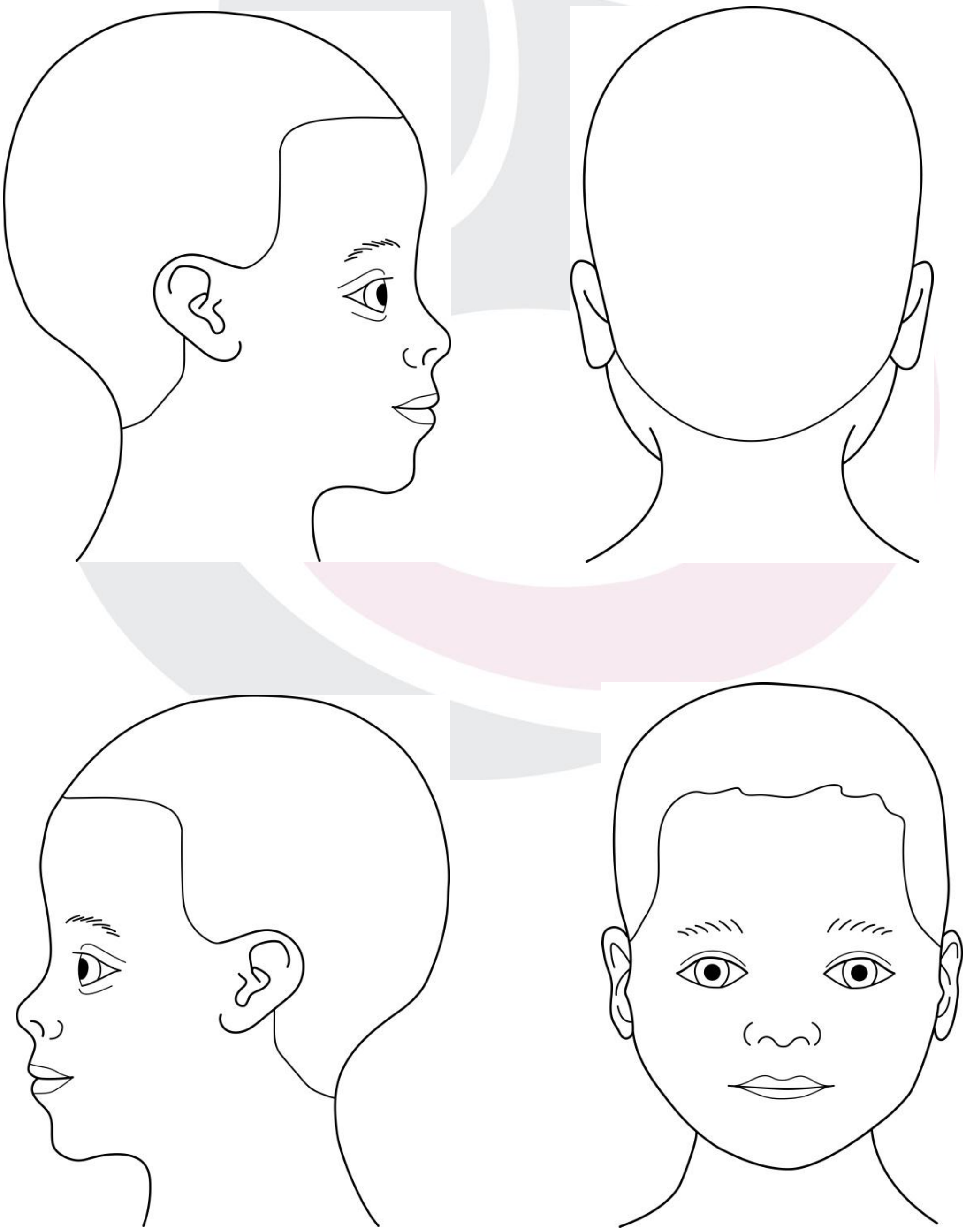

Statens Barnehus Kristiansand
Besøksadresse: Vestre Strandgate 26, 4611 Kristiansand
Postadresse: Postboks 135, 4662 Kristiansand

© Statens Barnehus Kristiansand

Tlf.: +47 99 25 71 61
Faks: +47 38 61 19 00
E-post: kristiansand@barnehuset.com
Foretaksnr.: NO 983 998 275
www.statensbarnehus.no

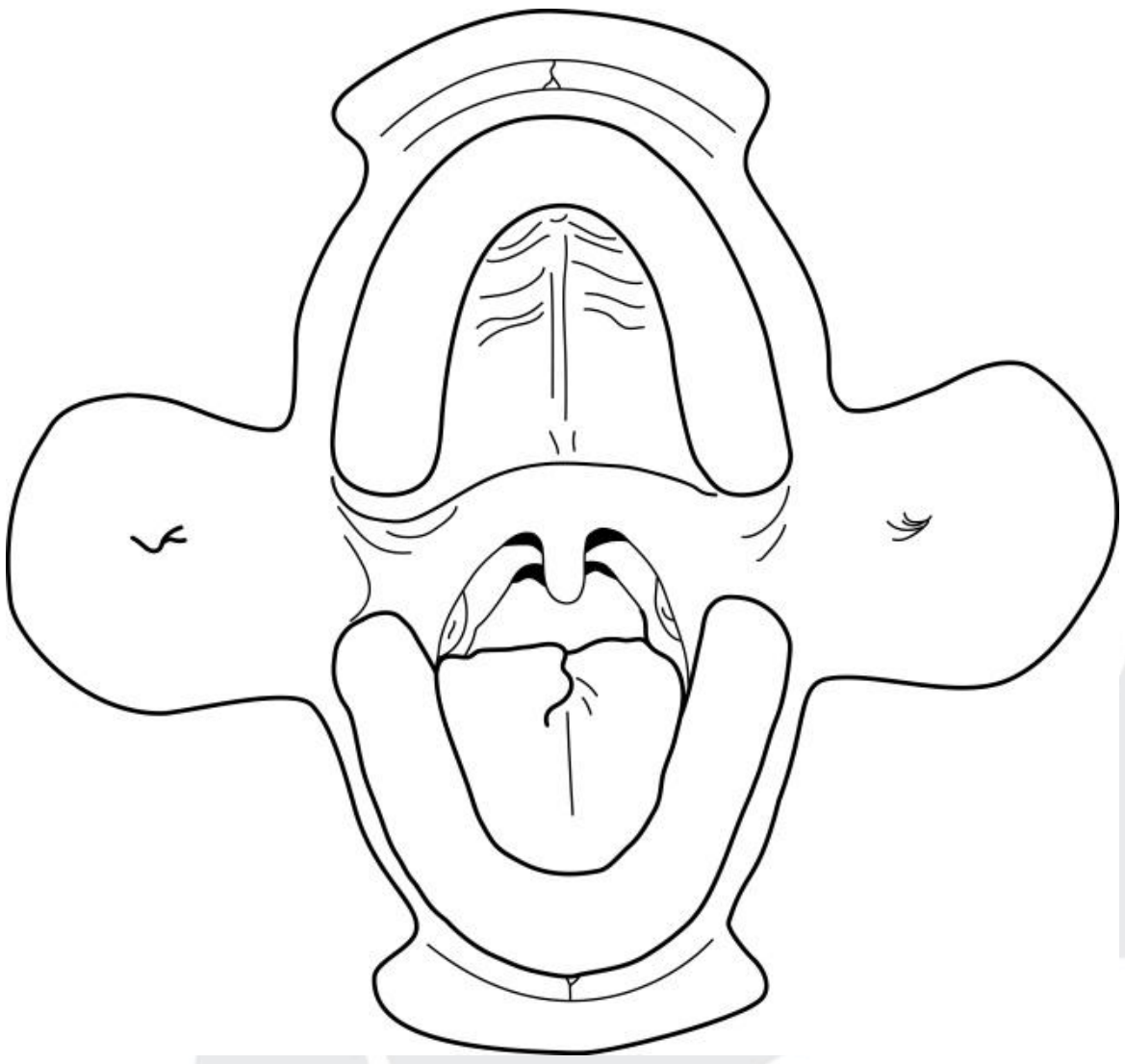

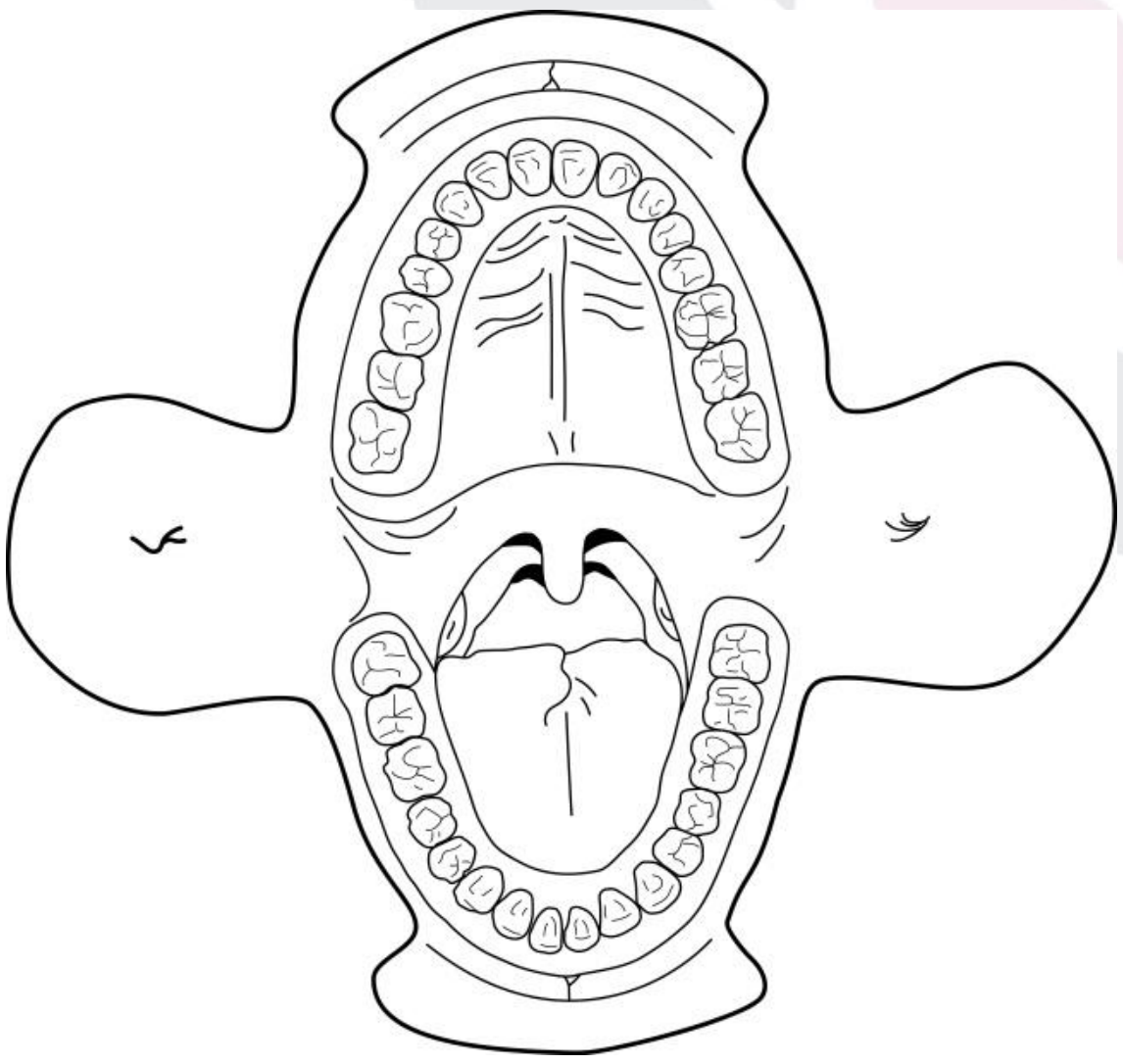

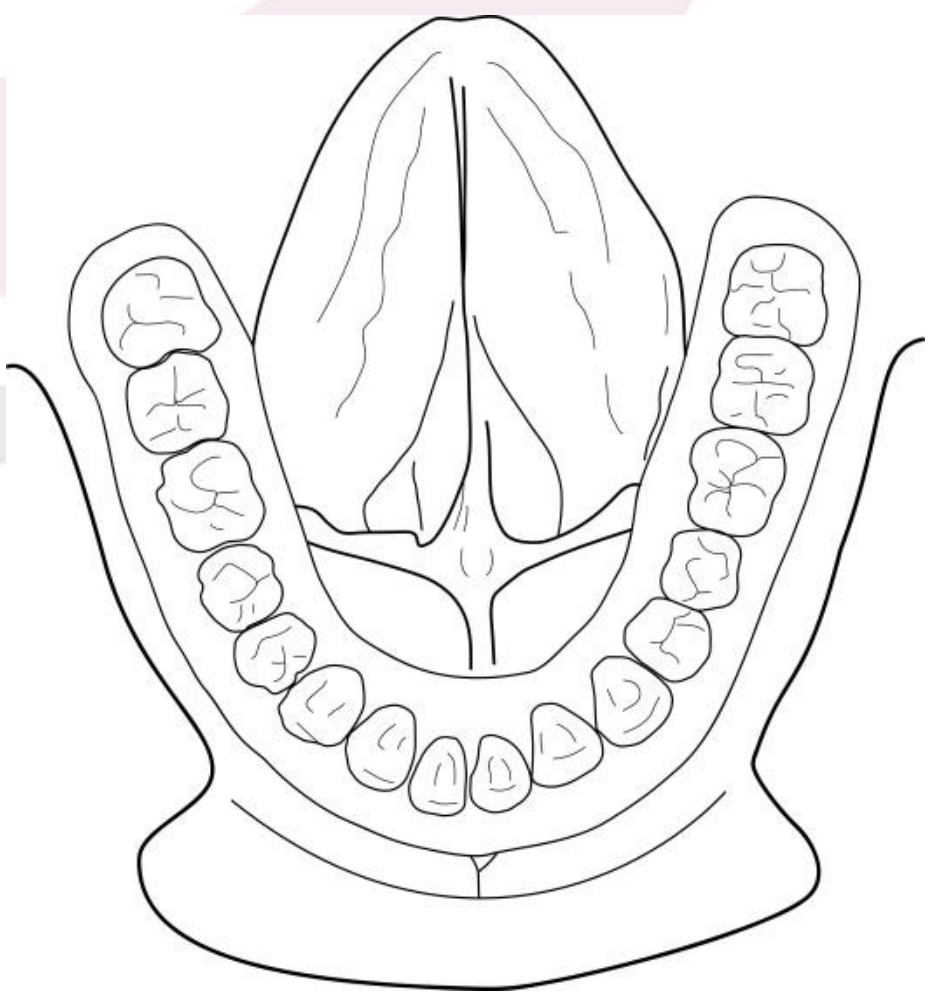

Statens Barnehus Kristiansand
Besøksadresse: Vestre Strandgate 26, 4611 Kristiansand
Postadresse: Postboks 135, 4662 Kristiansand

Tlf.: +47 99 25 71 61
Faks: +47 38 61 19 00
E-post: kristiansand@barnehuset.com
Foretaksnr.: NO 983 998 275
www.statensbarnehus.no

© Statens Barnehus Kristiansand

STATENS BARNEHUS
Et tverrfaglig kompetansehus
Kristiansand

Veiledning til skisse:
Manglende tenner (overkrysses(X)), rotrester markeres, protetiske erstatninger påtegnes og beskrives. Tilføy evt. anmerkninger som ikke fremgår av diagrammet (generelle og lokale periodontale forhold, okklusjonsforhold, kjeveleddsproblemer og andre funn som bør bemerkes).

Primære tannsett (melketannsett)

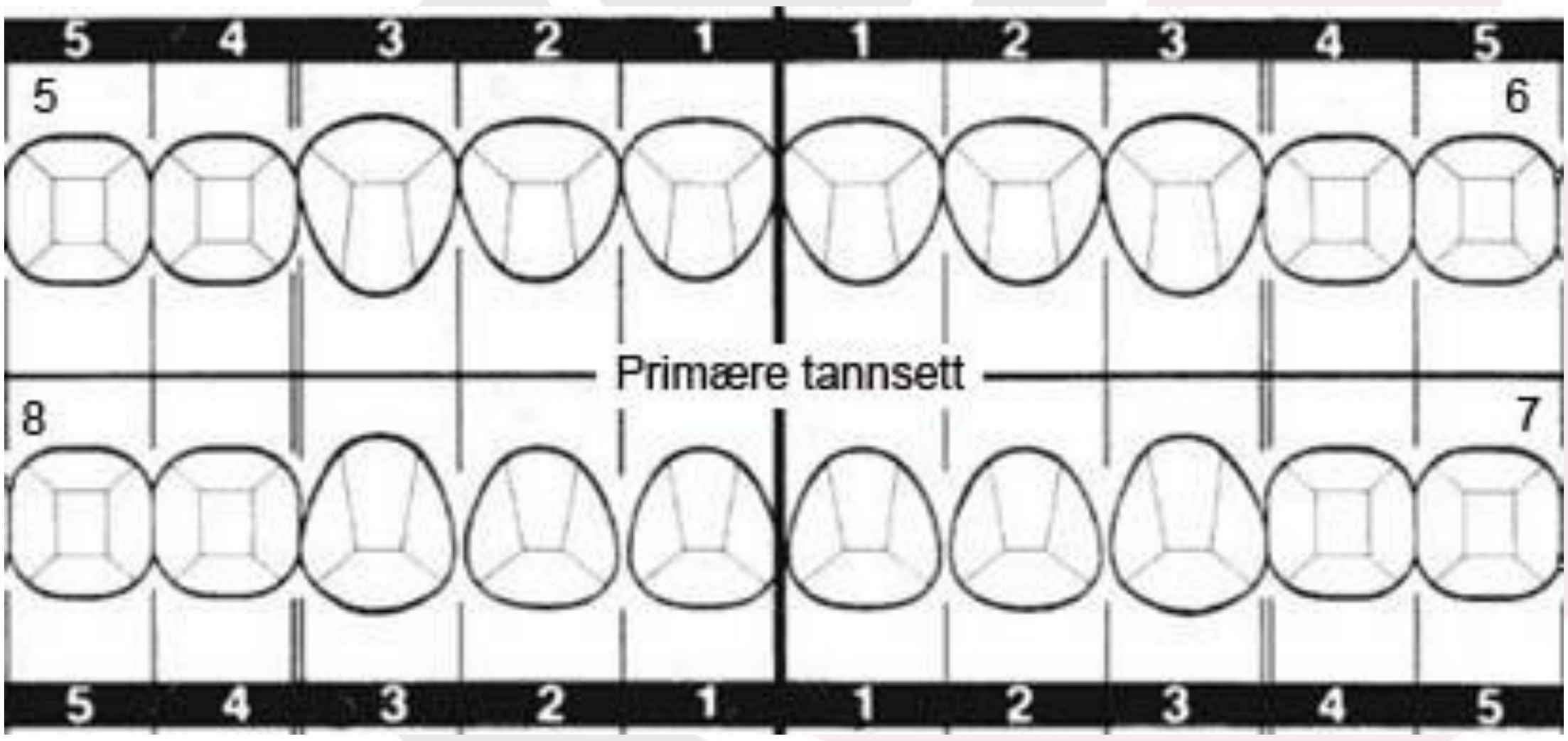

Permanent tannsett

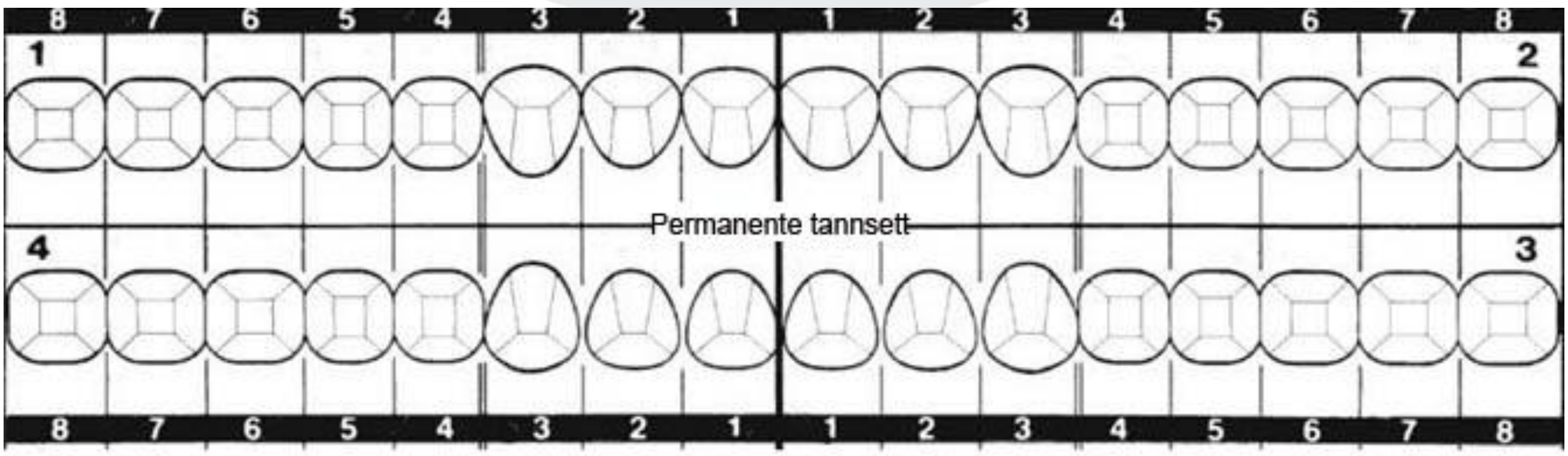

Ev. anmerkninger skrives her

Statens Barnehus Kristiansand
Besøksadresse: Vestre Strandgate 26, 4611 Kristiansand
Postadresse: Postboks 135, 4662 Kristiansand

Tlf.: +47 99 25 71 61
Faks: +47 38 61 19 00
E-post: kristiansand@barnehuset.com
Foretaksnr.: NO 983 998 275
www.statensbarnehus.no

© Statens Barnehus Kristiansand

STATENS BARNEHUS
Et tverrfaglig kompetansehus
Kristiansand

Oppdragsgiver:
Adresse *Skriv tekst her*
Kopi av erklæringen til den rettsmedisinke kommisjon Ja ☐ Nei ☐

Navnet på barnet/ungdommen: *Skriv tekst her*
Født: *Skriv tekst her*
Adresse: *Skriv tekst her*

Klinisk rettsodontologisk tannlegeerklæring

Mandat:
Beskriv mandatet her

Bakgrunn for undersøkelsen:
Skriv tekst her

Generell helsestatus:
Skriv tekst her

Klinisk tannlegeundersøkelse (ekstra og intraoral undersøkelse/beskrivelse av funn):
Skriv tekst her

Dokumentasjon:
Skriv tekst her

Konklusjon på tannlegeundersøkelsen:
Skriv tekst her

Vurdering:
Skriv tekst her

Hvilke råd er gitt til hvem:
Skriv tekst her

Vedlegg:
Bilder/prøvesvar

Tannlegeundersøkelsen er gjennomført av: *Skriv tekst her*

© Statens Barnehus Kristiansand
