# Supplementary material for: How physical and sexual abuse impacts oral health: a cross-sectional study from a Norwegian Children’s House
Source: BMC Oral Health. 2026 Jul 2;26:1841. doi: 10.1186/s12903-026-09143-3 (PMC13617796; doi:10.1186/s12903-026-09143-3)
Supplement: Supplementary file 3 [file 12903_2026_9143_MOESM3_ESM.docx]

**Not referred to forensic dental examinations**

(*n* = 1751)

**Duplicate records**

(*n* = 5)

**Missing record data**

(*n* = 37)

**Incomplete/ aborted examinations**	(*n* = 38)

ANALYSIS

SELECTION

**Children attending the NCH**

**2017-2021**

(*n* = 2159)

**Children referred for forensic dental examinations**

(*n* = 408)

**Children who underwent forensic dental examination**

(*n* = 371)

**Final study sample**

(*n* = 328)

IDENTIFICATION
